# Taeniasis in non-descript dogs in Ngorongoro, Tanzania: Prevalence and predisposing factors

**DOI:** 10.4102/ojvr.v83i1.1013

**Published:** 2016-05-24

**Authors:** Emmanuel S. Swai, Miran B. Miran, Ayubu A. Kasuku, Jahashi Nzalawahe

**Affiliations:** 1 Ministry of Livestock and Fisheries Development, Dar-es-Salaam, Tanzania; 2 Livestock Department, Ngorongoro District Council, Tanzania; 3 Department of Microbiology and Parasitology, Sokoine University of Agriculture, Tanzania

## Abstract

The prevalence of taeniasis was determined during the period January to April 2013 in a cross-sectional study of non-descript domestic dogs from the livestock–wildlife ecosystem of Ngorongoro, Tanzania. Taeniid eggs were determined by screening faecal samples using the formalin-ether sedimentation technique. Predisposing factors for dog infection were assessed in relation to demographic, husbandry and management data. Of the 205 faecal samples screened, 150 (73.2%) were positive for taeniid eggs. The prevalence of dogs harbouring taeniid eggs was 80%, 30.2% and 75.3% in the less than 1 year, 1–3 years and greater than 3 years of age groups, respectively. Age group and sex prevalence in dogs did not differ significantly (*P* > 0.05), although the females showed a marginally higher prevalence (73.8%) in comparison to the males (72.7%). Taeniid eggs were significantly more likely to be found in the faeces of dogs located in Waso (80.6%) and Endulen (75%) than in Malambo (63.2%, *P* < 0.05). The study revealed that dogs owned and raised by agro-pastoralists were at a lower risk of acquiring *Taenia* spp. infection (*P* = 0.001) than those that were raised by pastoralists. The majority of dog owners were not aware of the predisposing factors and the mode of transmission of taeniids. Dogs were frequently fed on viscera, trimmings and the heads of slaughtered animals, and they were not treated for parasitic infections. The findings of this study indicate that taeniasis is prevalent among non-descript dogs in Ngorongoro, underscoring the need for further research and active surveillance to better understand the transmission cycle of *Taenia* spp. in a wider geographical area in Tanzania.

## Introduction

Dogs have been reported to harbour a variety of gastro-intestinal parasites, some of which are of zoonotic importance (Craig & Macpherson 2000). The impact of infection is greater in sub-Saharan Africa because of the availability of a wide range of agro-ecological factors suitable for diversified hosts and parasite species (Folaranmi *et al.* 1984; Nonaka *et al.* 2011; Schandevyl, Mbundu & Sumbu 1987). Internal parasitism in domestic dogs (*Canis familiaris*) may result in loss of appetite and poor feed-conversion efficiency and predispose them to other pathogens or even death (Traub *et al.* 2005). The effects arising from the massive and sub-clinical infections include growth retardation, reduced weight gains and general ill-health (Soulsby 1982).

*Taenia multiceps* (Leske 1780) (syn. *Multiceps multiceps*), *Taenia hydatigena* and *Echinococcus granulosus* are worldwide cestode helminths that inhabit the small intestine of domestic dogs, wild dogs (*Lycaon pictus*), hyaenas (*Crocuta crocuta*), foxes (*Vulpes* spp.), coyotes (*Canis latrans*) and jackals (*Canis mesomelas, Canis aureus, Canis aductus*) (Scala *et al.* 2007). The definitive hosts for these cestode helminth species are members of the family Canidae. Many animals may serve as intermediate hosts; these include rodents, rabbits, horses, cattle, sheep and goats. The intermediate host becomes infected by ingesting proglottids or eggs passed in the faeces of the dog or wild carnivore in pastures or feeding areas. The definitive host becomes infected by ingesting the tissue of an infected intermediate host containing the metacestodes or larval stage of these worm species. Humans can also be affected by the intermediate host containing the larval stage of *T. multiceps* (*Coenurus cerebralis*), *T. hydatigena* (*Cysticercus tenuicollis*) and *E. granulosus* (cystic echinococcosis) (Sharma & Chauhan 2006). In the recent past, cases of coenurosis, hydatidosis and cysticercosis have been reported to occur in domestic small ruminants (sheep and goats) in various villages of Ngorongoro where the current study was undertaken (Ernest *et al.* 2009; Miran *et al.* 2015).

Surveys targeting helminth infections in dogs have been performed previously in some urban settings in Tanzania, but unfortunately no studies have ever been conducted in an agropastoral or pastoral setting where a considerable number of non-descript dogs can be found (Makene *et al.* 1996; Muhairwa, Nonga & Kusiluka 2008; Swai *et al.* 2010). Owing to the paucity of data on *Taenia* infections in dogs in rural areas, the present study was designed and implemented to estimate the prevalence of *Taenia* spp. infestation in dogs in Ngorongoro district, Tanzania. The presence of cestodes was confirmed by coprological examination, with the aim of generating baseline data and establishing the potential role of these parasites in causing coenurosis, cysticercosis and hydatidosis, often reported in sheep and goats in the study area.

## Material and methods

### Study area

**FIGURE 1:**
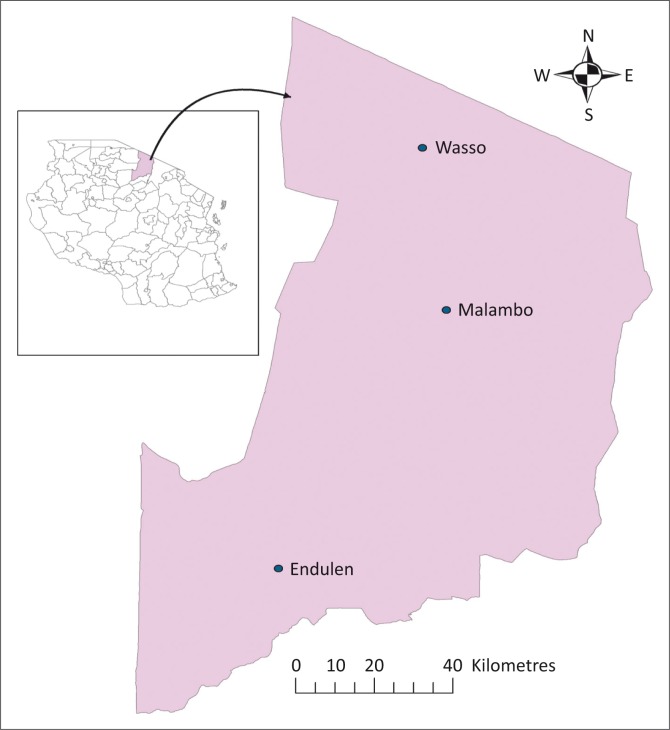
Map of Ngorongoro district showing the study villages. The insert is a map of Tanzania.

This study was conducted in Ngorongoro, the largest of the seven districts of Arusha region, northern Tanzania. The district lies between longitude 35’30° and 36’23° E and latitude 02’45° and 4’0° S and covers an area of 14 036 km^2^ (Figure 1). Geographically, the district is bordered by Kenya to the north, Serengeti National Park to the west, Longido and Monduli districts to the east and Karatu District to the south. Administratively, it is divided into 3 divisions, 21 wards and 43 registered villages and sub-villages. Currently the human population is estimated at 174 278, with a growth rate of 2.9% as opposed to the national growth rate of 3.0% per annum (URT 2012). The study sites were selected in collaboration with the government livestock extension and administration officers. The sites, commonly referred to as villages or wards, were selected on the basis of the geographical location (one from each of the three existing administrative divisions) and the presence of a high concentration of dogs and also of a slaughter slab where the slaughter of sheep and goats frequently takes place. The selected villages were Endulen, Malambo and Wasso. The study was conducted from January to April 2013.

### Study design and sample size

A cross-sectional study design was adopted and dogs selected for the study were examined and sampled once. Sample size estimation was based on a sampling frame of 2063 dogs distributed across the three selected villages using the formula developed by Martin, Meek and Willeberg (1987):

$$n=\frac{Z^{2}p(1-p)}{d^{2}}$$ [Eqn 1]

where: *n* = required sample size; *Z* = *Z*-value for a given confidence level (95% = 1.96); *d*^2^ = allowable error (0.05) and *p* = known or estimated prevalence.

The estimated prevalence was 15% based on the previous studies in Tanzania (Ernest *et al.* 2009). Therefore, the calculated sample size was 1.96^2^ x 0.15 x 0.85/0.05^2^ = 196. However, the practical working sample was raised to 205.

### Data collection

The breeds of dog kept were not known to most of the participating owners and therefore they were classified as non-descript. Data on the dogs’ demographic characteristics (age, sex, location) and history of deworming were collected. The ages of the examined dogs were stratified into four age-categories (< 1 yrs; 1–2 yrs; > 2 – < 3 yrs and > 3 yrs). Additional information pertaining to dog husbandry practices was sought by individual questionnaires administered to 121 livestock keepers who were also dog owners. At least 40 interviews were carried out per village. The selection criteria of an interviewee depended on convenience and/or owning dog(s). The information sought included disposal procedures of offal and/or infected brains condemned by the local meat inspector. These were categorised as (1) raw brain thrown to dogs, (2) thrown into the bush, (3) buried and fed dogs cooked brain from dead or slaughtered animals. Variables investigated included level of worm control practices, dog access to infected condemned offal and to raw brains and other trimmings thrown into the bush; and farming system where dog owners reside (agropastoral or pastoral).

#### Faecal sample collection and processing

Faecal samples from 205 domestic dogs (one sample per animal) from the villages where slaughter slabs are located (Wasso – highland, Endulen – medium and Malambo – lowland) were collected for laboratory analysis. Faecal samples were collected per rectum using fingers in glove, stored tightly closed, labelled and kept in a coolbox packed with an ice bag before transportation to the Tanzania Wildlife Research Institute’s (TAWIRI) Serengeti laboratory, where they were immediately examined or stored refrigerated (at 4 °C) for a maximum of one day before processing. Faecal samples were screened for the presence of taeniid eggs.

### Procedure or protocol

Faecal samples were analysed and interpreted using the formalin-ether sedimentation technique for fresh material (Ash & Orihel 1987). Briefly, approximately 1.0 g – 1.5 g of fresh faecal material was suspended in 10 mL 10% formalin in a 50 mL cup. The suspension was strained directly into a 15 mL conical centrifuge tube through two layers of wet gauze; the tube was filled almost to the brim with 0.85% saline solution and spun at 4000 × g for two minutes. In cases where the supernatant was still cloudy, it was discarded: the sediment was re-suspended and centrifuged again at 4000 × g using 0.85% saline solution. The supernatant was then discarded and the sediment re-suspended in 10% formalin by sharply flicking the bottom of the tube and more formalin was added to bring the total volume of the suspension to 10 mL. This was followed by adding 3 mL ether and the solution was shaken vigorously for about 3 seconds and then centrifuged at 5000 × g for 3 minutes.

The outcome formed four layers, namely, ether, debris that adheres to the wall of the tube, formalin and sediment layers. An applicator stick was inserted to ring and loosen the plug of debris and the three top layers were decanted and discarded. By using a glass pipette, a drop of saline was added and mixed with the sediment. Unstained wet mounts were prepared for examination for each sample and screened using a light microscope. The results were expressed either as positive or negative after microscopic examination based on the presence or absence of taeniid eggs.

### Data analysis

Data were coded and entered in a Microsoft Office Excel spreadsheet and analysed using Epi Info 7 software. Descriptive statistics were generated and presented as tables. The prevalence (P) of dogs harbouring taeniid egg(s) was calculated using the following formula:

$$p(\%)=\left(\frac{n}{N}\right)\times 100$$ [Eqn 2]

where *n* is the number of dogs diagnosed as having taeniid eggs at that point in time and *N* is the number of dogs at risk (examined) at that point in time (Thrusfield 2005). Associations between parasitism and categorical (host and management) factors were compared using the Chi-square test for independence. Fisher’s exact test was used when the numbers within categories were too small for the Chi-square test. In all the analyses, a value of *P* < 0.05 was considered significant.

## Ethical issues

Permission to carry out this study was granted by the Executive Director of Ngorongoro District and the research ethical committee of Sokoine University of Agriculture. Verbal consent was obtained from each participating farmer after explaining the purpose and importance of the study prior to starting the data collection. The operating procedures regarding the safety of the researchers, community and environment were strictly adhered to at all stages of sample collection, storage, transportation and processing.

## Results

### Descriptive statistics

Overall, 205 apparently healthy dogs belonging to 72 farmers were presented and faecal samples collected. Of the 205 dogs examined, 121 (59.1%) were males and 49 (40.9%) were females. Some male dogs (*n* = 16/93; 17.9%), mostly from the agro-pastoralist community, were reported to have been dewormed one year prior to the initiation of this study. The breed of dogs examined was defined as non-descript.

### Prevalence of taeniid eggs

Out of a total of 205, 150 (73.2%) were diagnosed as harbouring taeniid eggs at varying intensities. The proportions of dogs in each category and the mean faecal prevalence of taeniid eggs of each variable investigated during the study (*n* = 205) are given in Table 1.

**TABLE 1:** Proportions of dogs and prevalence of Taenia spp. infection in each category of each variable investigated during the study (*n* = 205).

Variable	Category	Number examined	Percentage (%)	Prevalence (%)	*χ*^2^	*P*-value
Sex	Females	84	40.90	62 (73.8)	0.86	0.0296
	Male	121	59.10	88 (72.7)		
Age	< 1 yr	30	15.40	24 (80.0)	3.70	0.2900
	1–2 yrs	66	33.80	48 (72.7)		
	> 2–3 yrs	24	12.30	14 (58.3)		
	> 3 yrs	85	43.60	64 (75.3)		
Village	Endulen	36	17.60	27 (75.0)	6.59	0.0370
	Malambo	76	37.10	48 (63.2)		
	Waso	93	45.40	75 (80.6)		
Owner production system	Agropastoral	93	45.40	37 (39.8)	14.67	0.000128
	Pastoral	112	54.60	74 (66.1)		
History of deworming	Yes	93	45.40	89 (95.7)	0.026	0.0408
	No	112	54.60	112 (100)		

### Factors associated with prevalence of taeniid egg infection

The factors influencing the prevalence of taeniid infection are given in Table 1. Host age was not found to be a significant factor with respect to the prevalence of taeniid eggs (*p* > 0.05), although eggs were detected more frequently in the young (≤ 1 year of age) than other categories. There was no significant difference in the prevalence of taeniid eggs between male (72.7%) and female (73.8%) dogs (*p* > 0.05). The production system was, however, found to be a significant factor with respect to the prevalence of taeniid (*p* < 0.05), with eggs being detected more frequently in dogs owned by pastoralists than agro-pastoralists. The prevalence rate was significantly higher in non-dewormed (*n* = 112, 100%) compared to dewormed dogs (*n* = 89, 95.7%; χ^2^ = 0.0266, *P* = 0.04086, Fisher exact test). At village level, Waso and Endulen had the highest proportion of dogs harbouring taeniid eggs (80.6% and 75% respectively) compared to Malambo (63%) (*df* = 2, χ*^2^* = 6.5, *P* = 0.0370).

## Predisposing factors for *Taenia* spp. infection

### Disposal of animal offal and infected organs or brain

The study found that during home slaughters 39.8% of respondents throw raw brain to dogs from clinically seen circling animals; 55.4% thrown into bush; 1.65% burned; 0.82% buried, and only 1.65% fed the brain from the animals that had died or had been slaughtered to their dogs after cooking (Table 2).

**TABLE 2:** Mode of disposal of condemned brains and other infected organs from slaughtered large and small ruminants.

Parameter	Level	No. of respondents (*n* = 121)	Percentage (%)
Thrown raw to dog(s)	Yes	49	39.80
	No	72	59.50
Thrown raw into bush	Yes	67	55.40
	No	54	44.60
Burned	Yes	2	1.65
	No	119	98.30
Fed to dog after cooking	Yes	2	1.65
	No	119	98.30
Buried	Yes	1	0.82
	No	6	99.20

### Predisposing factors associated with the acquisition of *Taenia* spp.

The study has also shown that predisposing factors associated with the acquisition of *Taenia* spp. are dogs left free all the time and some shepherd dogs (98.2%) and a lack of worm control in dogs (93.6%) (Table 3). At the same time, it was found that the deworming of domestic dogs was influenced by the farming system: in pastoral areas 100% of the respondents had never dewormed their dogs before, whereas only 17.9% in agropastoral areas where veterinary services are available sometimes dewormed their dogs, although not routinely.

**TABLE 3:** Predisposing factors for *Taenia* spp. infection in dogs in the Ngorongoro district.

Predisposing factors	Level	No. of respondents (*n* = 110)	Percentage (%)
Lack of worm control in dog(s)	Yes	103	93.6
	No	7	6.4
Domestic dogs scavenging	Yes	10	9.1
	No	100	90.9
Dogs left free all time and herding	Yes	108	98.2
	No	2	1.8

## Discussion

The microscopic faecal examination showed that the prevalence of *Taenia* spp. infection was high in the study area. This finding is in agreement with the results of other researchers that *Taenia* spp. are common parasitic zoonotic intestinal tapeworms in dogs worldwide (Anosike *et al.* 2004; Davoust *et al*. 2008; Minnaar, Krecek & Fourie 2002; Muhairwa *et al*. 2008). The overall prevalence of 73.3% of *Taenia* spp. eggs in the dogs in this study shows that there were frequent infections of our indigenous non-descript dogs with *Taenia* spp., and these species are most probably highly prevalent in the study area, reflecting the poor husbandry practices and gross lack of improvement in the country’s animal health management programmes.

The high prevalence of taeniid eggs in domestic dogs indicates a high level of contamination of pastures/environment with the eggs in addition to contamination with faeces of infected wild carnivores, which was found to be highly prevalent in the area (M.B. Miran, personal communication 2015). It was found that worm control in dogs was not a common practice in the study area, with 93.6% never having dewormed their dogs. A small proportion (6.4%) of dog owners did deworm their dogs, although not routinely.

Reports of *Taenia* spp. infection in some countries in Africa and other tropical to sub-tropical regions revealed a lower prevalence of taeniid eggs: 18.3% in Ethiopia (Degefu, Tefera & Yohannes 2011), 13% in Zambia (Nonaka *et al.* 2011), 9.4% in Zaria, Nigeria (Folaranmi *et al.* 1984) and 2.7% in the urban areas of Kinshasa, Zaire (now Democratic Republic of the Congo) (Schandevyl *et al.* 1987). The variability in the prevalence reported could be attributed to differences in the production system, the diagnostic procedures employed, the relative absence or presence of herbivore hosts, the level of immunity and husbandry practices such as the regular deworming of dogs.

Dogs less than one year old and between one and 2 years old (80% and 72.7%, respectively) were harbouring more taeniid eggs than dogs older than 2–3 years (58.3%). The reasons for the lower prevalence of the eggs in this age group may be explained by the presence of an acquired immunity (Gemmell, Lawson & Roberts 1987). There could be a number of reasons for the relatively high prevalence of taeniid eggs in one year old and younger dogs, including low immunity (Soulsby 1982).

The results obtained in this study revealed that the difference in the prevalence of taeniid eggs in male and female dogs was not statistically significant. This concurs with reports by Yacob *et al.* (2007) and Degefu *et al.* (2011) conducted in Debre Zeit and Jimma (Ethiopia), respectively.

Dogs raised in pastoral areas were significantly associated with a higher prevalence than those in agro-pastoral settings. The frequent contacts and probably a general lack of slaughter points in most pastoral areas could lead to dogs having wider access of infected offal or brain tissues due to the gross lack of disposal pits.

The present study employed a formalin-ether sedimentation method that is believed to be less sensitive than tests of higher sensitivities and specificities such as PCR (Christie *et al.* 2011; Scandrett & Gajadhar 2004; Trachsel, Deplazes & Mathis 2007). Based on the cross-sectional study design and test employed, the authors would anticipate lower or higher than the true prevalences of taeniid eggs. However, the observed high prevalence detected in this survey suggests that the infection rates of *Taenia* spp. are high in the current study areas. Since the eggs of the various *Taeni*a spp. cannot be distinguished microscopically from one another or from those of *Echinococcus* spp., other diagnostic procedures should be employed (Allan *et al.* 2003). There is a need to develop a diagnostic method that would detect *Taenia* infections in dogs at species level.

At all three slaughter slabs where the study was conducted, each slab had a condemnation pit for the disposal of the unfit offal and/or carcasses. However, it was observed that these were not adequately used, especially during livestock market days, as meat inspectors do not ensure proper disposal of condemned organs. As a result, some organs or trims were simply thrown away. These pits are not fenced and they are too shallow to restrict access by scavenging dogs.

## Conclusion

This study established high prevalence of taeniid eggs in dogs, which is likely to be associated with lack of knowledge regarding the predisposing factors, gross lack of helminth control measures (deworming) and high complexity of interaction between definitive hosts and feeding habits of dogs of condemned offal from intermediate hosts such as slaughtered goats and sheep. Furthermore, extensive molecular and epidemiological studies are needed to ascertain, among other things, whether there is genetic variation among *Taenia* spp. eggs that is microscopically indistinguishable by the screening method used. Moreover, the transmission cycle between domestic species and wildlife at the livestock–wildlife interface is not well understood. This therefore calls for additional research and improved surveillance that should lead to more appropriate control measures of the parasite and the development of a simple field diagnostic method for detecting *Taenia* spp. infection in both definitive and intermediate hosts. Finally, there is a need for better public awareness of the potential human health hazard posed by inadvertently ingesting *Taenia* eggs carried by infected domestic and wild carnivores.
